# Crystallographic Snapshots of Pre- and Post-Lanthanide Halide Hydrolysis—Reaction Products Captured by the 4‑Amino-1,2,4-triazole Ligand

**DOI:** 10.1021/acs.cgd.5c00835

**Published:** 2025-07-19

**Authors:** Volodymyr Smetana, Geetha Bolla, Ethan A. Hiti, Hannah Wineinger, Anja-Verena Mudring, Robin D. Rogers

**Affiliations:** † Department of Chemistry, 228871 University of Wyoming, 1000 E, University Ave, Laramie, Wyoming 82071, United States; ‡ Department of Chemistry & Biochemistry, The University of Alabama, Tuscaloosa, Alabama 35401, USA; § Department of Biological and Chemical Engineering and iNANO, 1006 Aarhus University, 8000 Aarhus C, Denmark; ∥ Department of Physics, 8075 Umeå University, 90187 Umeå, Sweden

## Abstract

Reactions of lanthanide­(III) chloride salts with 4-amino-1,2,4-triazole (4-NH_2_-1,2,4-Triaz) in azole melts have led to the isolation of both hydrolysis and nonhydrolysis products in the same synthesis, with the inclusion of a variety of ligands, anions, and water, allowing us to capture crystallographic snapshots of different forms and intermediate hydrolysis fragments. The structural studies reported here include anhydrous and hydrated nonhydrolyzed complexes, which were isolated alongside hydrolysis products, giving oxide/hydroxide lanthanide­(III) dimers, tetramers, and ultimately hexamers. The compounds isolated include [Nd_2_Cl_6_(μ_2_-4-NH_2_-1,2,4-Triaz)_4_(4-NH_2_-1,2,4-Triaz)_2_], [Ce_2_Cl_4_(μ_2_-Cl)_2_(μ_2_-4-NH_2_-1,2,4-Triaz)_4_]_n_, [Ce_2_(μ_2_-Cl)_4_(μ_2_-OH)_2_(μ_2_-4-NH_2_-1,2,4-Triaz)_2_]_n_, [Ln_4_Cl_4_(μ_2_-Cl)_4_(μ_3_-OH)_4_(μ_2_-4-NH_2_-1,2,4-Triaz)_4_]_n_·2*n*H_2_O (Ln = Ce, Nd), and [Ce_6_Cl_6_(μ_6_-O_0.5_)­(μ_3_-Cl_0.5_)_4_(μ_3_-Cl_0.75_)_3_(μ_3_-OH)_0.75_(μ_2_-4-NH_2_-1,2,4-Triaz)_12_((OH_2_)_0.25_)_2_]_2_[CeCl_6_]­[Cl_9_]·*x*H_2_O. In all complexes, all lanthanide atoms are pairwise connected via one or more 4-NH_2_-1,2,4-Triaz ligands and sometimes additional Cl^–^ anions.

## Graphical abstract

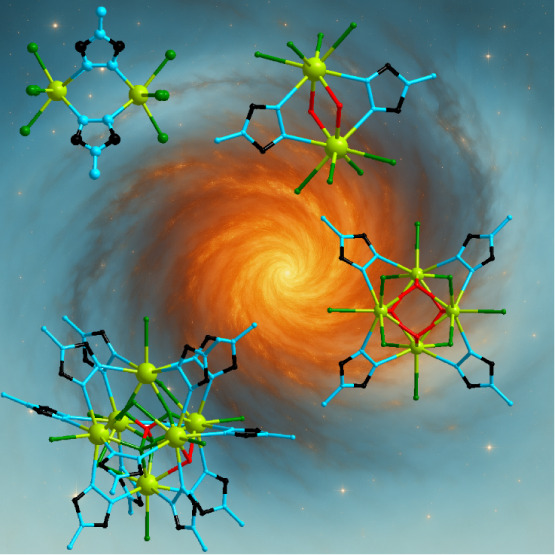

## Introduction

Interactions between f-elements and moderately soft donors have been intensively investigated due to their high importance in critical applications, particularly nuclear waste utilization. N-donor reagents show high selectivity toward 4*f*/5f elements and can be applied for lanthanide/actinide separations.^1^ Understanding the mechanisms of these interactions, development of the synthetic procedures, and consequently, extractant optimization has always been achieved through extensive explorations over the past decades.^2–5^ Among multiple approaches, ionic liquids (ILs) have been considered a universal tool that can act as a solvent, reaction medium, and a selective coordination agent with any required functional groups by demand, particularly N-donors.^6–11^ Since N-heterocycles have already been shown as promising actinide-selective ligands,^4^ their ionic liquids are of enhanced interest. As direct access to Pu­(III/IV) salts is complicated, lanthanide proxies, i.e., Ce­(III/IV) and Nd­(III) analogs can be used instead to gain some understanding of their behavior.^9–11^

One difficulty that has arisen, particularly in nuclear waste remediation, is that uncontrolled hydrolysis results in unpredictable products, including precipitates and colloids, which interfere with modern separation processes. Although hydrolysis reactions of the actinides have been intensively studied,^12^ their reproducibility and versatility still depend on the huge variability of independent criteria such as solubility, pH, nature of the ligands, and reaction medium, etc., offering an endless field for exploration. We would also note that little attention has been paid to hydrolysis in the presence of N-donor ligands.

In our own studies of ionic liquid or ionic-liquid-like azole and azolium ligands, we have also found hydrolysis to be a concern. We were especially intrigued, however, by the serendipitous behavior of the 4-amino-1,2,4-triazole (4-NH_2_-1,2,4-Triaz) ligand allowing the isolation of the unprecedented hexanuclear Ce­(III) complex, [Ce_6_(μ_3_-O)_4_(μ_3_-OH)_2_(μ_3_-Cl)_2_Cl_6_(μ_2_-4-NH_2_-1,2,4-Triaz)_12_]·7H_2_O.^13^ Our subsequent study of similar reaction conditions led to the isolation of an analogous Nd­(III) complex, [Nd_6_(μ_3_-OH)_8_Cl_6_(μ_2_-4-NH_2_-1,2,4-Triaz)_12_]­[Cl_4_]·2H_2_O.^14^ It seemed to indicate our chosen ligand, 4-NH_2_-1,2,4-Triaz, could hold bridged Ln­(III) ions together in solution while mixtures of ligands, anions, and water generated a number of disparate structural results, often heavily disordered.^13^ Our most recent paper even showed that the same structural form (unit cell, symmetry, etc.) can apparently form from what are really fragments of the hexamer,^14^ where structural studies of [Eu_6_(μ_6_-Cl)_0.23_(μ_3_-O_0.77_)_4_(μ_3_-O)_2.6_(μ_3_-Cl)_0.4_Cl_6_(μ_2_-4-NH_2_-1,2,4-Triaz)_12_] and [Ho_6_(μ_6_-Cl)_0.21_(μ_3_-O_0.79_)_4_(μ_3_-OH)_2_Cl_6_(μ_2_-4-NH_2_-1,2,4-Triaz)_12_]­[Cl]_3.4_ indicated the very incomplete formation of either an Ln_6_X_9_ or Ln_6_X_8_ (X = O^2–^, OH^–^, Cl^–^) core.

We have thus further explored what structural entities we could “capture” with this simple synthetic strategy, as we apparently have still only started to uncover a plethora of different forms and intermediate fragments. While additional experimental techniques (e.g., spectroscopic tracking of solution dynamics or kinetic measurements) could further deepen the analysis, the ability to isolate and structurally characterize a wide variety of intermediates already provides compelling evidence for the pathways proposed. The internal consistency and diversity of isolated structures under identical or minimally varied conditions suggest that these observations are not isolated anomalies but rather reflect intrinsic tendencies of lanthanide chemistry in such donor-rich environments and, therefore, offers valuable insight into f-element coordination chemistry despite experimental challenges in obtaining single-phase products. Here we expand the number of structural results with examples of anhydrous and hydrated nonhydrolyzed complexes that were isolated alongside hydrolysis products, giving oxide/hydroxide dimers, tetramers, and ultimately hexamers, suggesting we are crystallizing intermediates before, during, and after hydrolysis.

Specifically, we compare our previously reported results, namely the isolation of [Ce_2_Cl_6_(μ_2_-4-NH_2_-1,2,4-Triaz)_4_(4-NH_2_-1,2,4-Triaz)_2_], [Ce_2_Cl_2_(μ_2_-4-NH_2_-1,2,4-Triaz)_4_(OH_2_)_8_]­Cl_4_·4H_2_O, and [Ce_6_(μ_3_-O)_4_(μ_3_–OH)_2_(μ_3_-Cl)_2_Cl_6_(μ_2_-4-NH_2_-1,2,4-Triaz)_12_]·7H_2_O,^13^ with newly isolated unhydrolyzed [Nd_2_Cl_6_(μ_2_-4-NH_2_-1,2,4-Triaz)_4_(4-NH_2_-1,2,4-Triaz)_2_] and [Ce_2_Cl_4_(μ_2_-Cl)_2_(μ_2_-4-NH_2_-1,2,4-Triaz)_4_]_n_ and a hydrolyzed dimer [Ce_2_(μ_2_-Cl)_4_(μ_2_-OH)_2_(μ_2_-4-NH_2_-1,2,4-Triaz)_2_]_n_, tetramer [Ln_4_Cl_4_(μ_2_-Cl)_4_(μ_3_-OH)_4_(μ_2_-4-NH_2_-1,2,4-Triaz)_4_]_n_·2nH_2_O (Ln = Ce, Nd), and new complex hexamer [Ce_6_Cl_6_(μ_6_-O_0.5_)­(μ_3_-Cl_0.5_)_4_(μ_3_-Cl_0.75_)_3_(μ_3_–OH)_0.75_(μ_2_-4-NH_2_-1,2,4-Triaz)_12_((OH_2_)_0.25_)_2_]_2_[CeCl_6_]­[Cl_9_]·*x*H_2_O. These results add important data, which ultimately will allow determination of the complex mechanism of hydrolysis product formation of Pu­(III) salts all the way from smaller fragments to the ubiquitous hexamer^15^ capturing snapshots of what is happening in solution.

## Experimental Section

### Reagents

The metal salts NdCl_3_·7H_2_O and CeCl_3_ *·*7H_2_O were obtained from Strem Chemicals (Newburyport, Massachusetts) at 99.9% purity and were used as received. 4-Amino-1,2,4-triazole (4-NH_2_-1,2,4-Triaz) and 1,2,3-triazole (1,2,3-HTriaz) were purchased from Sigma-Aldrich, Inc. (St. Louis, Missouri) at 99% purity and were used as received. Deionized (DI) water used in the reactions was obtained from a commercial deionizer (Culligan, Northbrook, IL, USA) with a specific resistivity of 16.82 MΩ·cm at 25 °C.

#### Synthesis of [Nd_2_Cl_6_(μ_2_-4-NH_2_-1,2,4-Triaz)_4_(4-NH_2_-1,2,4-Triaz)_2_] and [Nd_4_Cl_4_(μ_2_-Cl)_4_(μ_3_-OH)_4_(μ_2_-4-NH_2_-1,2,4-Triaz)_4_]_n_·2*n*H_2_O

4-NH_2_-1,2,4-triazole (168 mg, 2 mmol, 7.4 equiv) and 1,2,3-triazole (210 mg, 3 mmol, 11.1 equiv) were added to a 1.5-dram vial and heated to 120 °C for 15–20 min to yield a colorless liquid. NdCl_3_·7H_2_O (103, mg, 0.27 mmol, 1 equiv) was added to another 1.5-dram vial with H_2_O (0.4 mL, 22.22 mmol, 82 equiv) and heated to 120 °C for 20 min until a transparent solution was formed. Both vials were kept at room temperature for 5 min, then the metal salt solution was added to the azole mixture, followed by heating to 120 °C for 4 h with stirring. The resulting mixture was placed in a sand bath for crystallization at 90–95 °C. After 8 weeks, pink block- and needle-shaped crystals were observed. Single crystal X-ray diffraction (SCXRD) examination revealed the needle crystals to be [Nd_4_Cl_4_(μ_2_-Cl)_4_(μ_3_-OH)_4_(μ_2_-4-NH_2_-1,2,4-Triaz)_4_]_*n*_·2*n*H_2_O and the block crystals to be [Nd_2_Cl_6_(μ_2_-4-NH_2_-1,2,4-Triaz)_4_(4-NH_2_-1,2,4-Triaz)_2_].

#### Synthesis of [Ce_2_Cl_4_(μ_2_-Cl)_2_(μ_2_-4-NH_2_-1,2,4-Triaz)_4_]_*n*_ and [Ce_2_(μ_2_-Cl)_4_(μ_2_-OH)_2_(μ_2_-4-NH_2_-1,2,4-Triaz)_2_]_*n*_

4-NH_2_-1,2,4-triazole (268 mg, 3.19 mmol, 9.9 equiv) and 1,2,3-triazole (428 mg, 6.2 mmol, 19.3 equiv) were added to a 1 dram vial and heated to 120 °C for 15–20 min to obtain a colorless liquid. A solution of CeCl_3_·7H_2_O (115 mg, 0.32 mmol, 1 equiv) in H_2_O (0.5 mL, 27 mmol, 85 equiv) was placed in another 1 dram vial and heated to 120 °C for 20 min until a transparent solution was observed. Both vials were kept at room temperature for 5 min, then the metal salt solution was added to the azole mixture, followed by heating to 120 °C for 4 h with stirring. The resulting mixture was placed in a sand bath for crystallization at 90 °C. After 8 weeks, colorless block crystals of both compounds were confirmed by SCXRD.

#### Synthesis of [Ce_4_Cl_4_(μ_2_-Cl)_4_(μ_3_-OH)_4_(μ_2_-4-NH_2_-1,2,4-Triaz)_4_]_*n*_·2*n*H_2_O

4-NH_2_-1,2,4-triazole (256 mg, 3.04 mmol, 11.1 equiv) and 1,2,3-triazole (228 mg, 3 mmol, 11.1 equiv) were placed in a 1 dram vial and heated at 120 °C for 20 min to melt the azoles. A solution of CeCl_3_·7H_2_O (98, mg, 0.27 mmol, 1 equiv) in H_2_O (0.5 mL, 27 mmol, 99 equiv) was prepared in another 1 dram vial by heating at 120 °C for 20 min until a transparent solution appeared. The azole melt and salt solution were kept are room temperature for 5 min, the salt solution added to the azoles and the resulting mixture was heated for 4 h at 120 °C with stirring. The resulting mixture was placed in a sand bath for crystallization at 90–95 °C. After 8 weeks, colorless block crystals were observed and confirmed by SCXRD to be [Ce_4_Cl_4_(μ_2_-Cl)_4_(μ_3_-OH)_4_(μ_2_-4-NH_2_-1,2,4-Triaz)_4_]_*n*_·2*n*H_2_O (isostructural with the Nd complex).

#### Synthesis of [Ce_6_Cl_6_(μ_6_-O_0.5_)­(μ_3_-Cl_0.5_)_4_(μ_3_-Cl_0.75_)_3_(μ_3_-OH)_0.75_(μ_2_-4-NH_2_-1,2,4-Triaz)_12_((OH_2_)_0.25_)_2_]_2_[CeCl_6_]­[Cl_9_]·*x*H_2_O

4-NH_2_-1,2,4-triazole (255 mg, 3.03 mmol, 20 equiv) and 1,2,3-triazole (223 mg, 3.1 mmol, 20 equiv) were placed in a 1.5-dram vial and heated to 120 °C for 15–20 min to yield a colorless liquid. A solution of CeCl_3_·7H_2_O in 0.5 mL (27 mmol) water was added to another 1.5 dram vial to make a salt solution. The metal salt solution was added to the azole vial at room temperature and the reaction mixture was placed in a sand bath at 100–110 °C for reaction and crystallization. After 2 weeks a milky-colored solution was observed with a gray precipitate and the reaction mixture was cooled to room temperature and centrifuged. The supernatant was placed in a new vial and placed back in the sand bath at 100–110 °C. After 18 weeks pale-yellow crystals were observed and confirmed by SCXRD to be [Ce_6_Cl_6_(μ_6_-O_0.5_)­(μ_3_-Cl_0.5_)_4_(μ_3_-Cl_0.75_)_3_(μ_3_–OH)_0.75_(μ_2_-4-NH_2_-1,2,4-Triaz)_12_((OH_2_)_0.25_)_2_]_2_[CeCl_6_]­[Cl_9_]·*x*H_2_O.

### Single Crystal X-Ray Diffraction (SCXRD) Analyses

SCXRD data for all compounds except [Ce_6_Cl_6_(μ_6_-O_0.5_)­(μ_3_-Cl_0.5_)_4_(μ_3_-Cl_0.75_)_3_(μ_3_–OH)_0.75_(μ_2_-4-NH_2_-1,2,4-Triaz)_12_((OH_2_)_0.25_)_2_]_2_[CeCl_6_]­[Cl_9_]·*x*H_2_O were collected on a Rigaku XtaLAb Synergy-R DW-single crystal X-ray diffractometer equipped with a Hypix 6000HE detector and MoKα radiation (Rigaku Corporation, Tokyo, Japan). The latter compound was measured on a Bruker D8 Venture Duo X-ray diffractometer equipped with a CMOS IμS 3.0 Mo source (λ = 0.71073 Å) set at 50 kV and 1.4 mA and a PHOTON detector. Single crystals were hand-picked with needles coated with Paratone^©^ oil and put on glass slides from which specimens were selected, placed in the single crystal loop holders, and then cooled to 100 K under a cold stream of nitrogen using an Oxford cryostat (Oxford Cryosystems, Oxford, UK). Hemispheres of data out to a resolution of at least 0.80 Å were collected using φ and ω scans. Unit cell analysis, initial structure solution and further refinements have been performed with SHELXT^15^ and SHELXL^16^ within the APEX4 software suite (Bruker AXS). Non-hydrogen atoms were located from the difference map and refined anisotropically. Hydrogen atoms bonded to heavy atoms were placed in calculated positions, and their coordinates and displacement parameters were constrained to ride on the carrier atoms. Platon Squeeze^17^ algorithms were employed to treat disordered solvent molecules impossible to locate on the differential Fourier maps. For the heavily disordered compound, occupancies of all positions were initially refined based on the observed Fourier maps. Some occupancies were then fixed to maintain reasonable distances/occupation and charge balance. This was particularly important for the μ_3_ positions of the octahedral complex. Short contact analysis and structure visualization were done with Diamond software.^18^

## Discussion

To capture crystallographic snapshots of complex f-element hydrolysis starting with lanthanide salts, we use simple synthetic approaches, so that these techniques can be applied to transuranic isotopes in the near future. For this, we started with a mixture of the low melting 4-amino-1,2,4-triazole (mp ca. 84–86 °C) and 1,2,3-triazole (mp ca. 23–25 °C) and prepared a melt. Although this mixture does not ionize to form an ionic liquid, it behaves much like an ionic liquid, the liquid melts acting as a source of both ligands and solvent for metal ions. One benefit of this approach we have found in working with hydrated f-element starting salts and even aqueous solutions of these salts, is that the ILs or azole melts often displace water from the metal coordination sphere,^6^ something we observe in all of the compounds reported here.

Adding aqueous chloride Ce­(III) and Nd­(III) solutions to the azole melt and long-term heating in a sand bath led to the crystallization and structural characterization of [Nd_2_Cl_6_(μ_2_-4-NH_2_-1,2,4-Triaz)_4_(4-NH_2_-1,2,4-Triaz)_2_], [Ce_2_Cl_4_(μ_2_-Cl)_2_(μ_2_-4-NH_2_-1,2,4-Triaz)_4_]_n_, [Ce_2_(μ_2_-Cl)_4_(μ_2_-OH)_2_(μ_2_-4-NH_2_-1,2,4-Triaz)_2_]_n_, [Ln_4_Cl_4_(μ_2_-Cl)_4_(μ_3_-OH)_4_(μ_2_-4-NH_2_-1,2,4-Triaz)_4_]_*n*_·2*n*H_2_O (Ln = Ce, Nd), and [Ce_6_Cl_6_(μ_6_-O_0.5_)­(μ_3_-Cl_0.5_)_4_(μ_3_-Cl_0.75_)_3_(μ_3_-OH)_0.75_(μ_2_-4-NH_2_-1,2,4-Triaz)_12_((OH_2_)_0.25_)_2_]_2_[CeCl_6_]­[Cl_9_]·*x*H_2_O. We note that unhydrolyzed/anhydrous compounds are often isolated from the same reaction mixture. Here both [Nd_2_Cl_6_(μ_2_-4-NH_2_-1,2,4-Triaz)_4_(4-NH_2_-1,2,4-Triaz)_2_] and hydrolyzed [Nd_4_Cl_4_(μ_2_-Cl)_4_(μ_3_-OH)_4_(μ_2_-4-NH_2_-1,2,4-Triaz)_4_]_*n*_·2*n*H_2_O and [Ce_2_Cl_4_(μ_2_-Cl)_2_(μ_2_-4-NH_2_-1,2,4-Triaz)_4_]_*n*_ and [Ce_2_(μ_2_-Cl)_4_(μ_2_–OH)_2_(μ_2_-4-NH_2_-1,2,4-Triaz)_2_]_*n*_ were isolated from the same reaction vessels.

We had previously isolated from a similar reaction [Ce_2_Cl_6_(μ_2_-4-NH_2_-1,2,4-Triaz)_4_(4-NH_2_-1,2,4-Triaz)_2_] and reported its crystal structure,^13^ suggesting that the azole ligand began organizing (bridging) Ln­(III) ions in solution, even prior to any hydrolysis. In our current study, we isolated an isostructural product of Nd­(III), the anhydrous [Nd_2_Cl_6_(μ_2_-4-NH_2_-1,2,4-Triaz)_4_(4-NH_2_-1,2,4-Triaz)_2_] (*P*1̅ , *a* = 9.1751(3), *b* = 10.1310(4), *c* = 17.4113(3) Å, α = 97.008(2), β = 92.681(2), γ = 90.710(3)°, *V* = 1604.31(9) Å^3^, Z = 2). Interestingly, in our current synthesis, this anhydrous product was isolated in the same reaction vessel as a lower-order hydrolysis product as noted above, revealing adjacent steps of the ongoing hydrolysis.

In [Nd_2_Cl_6_(μ_2_-4-NH_2_-1,2,4-Triaz)_4_(4-NH_2_-1,2,4-Triaz)_2_], the Nd atoms are connected via four azole bridges and each of them is additionally coordinated by three Cl^–^ and an extra terminal monodentate azole ligand (Figure 1). Neither coordinated nor solvent water could be observed in the crystal lattice. We have previously described the structure of the Ce­(III) complex^13^ and here would note that the major difference, as expected, lies in the Ln–Ln separations reflecting the lanthanide contraction—4.6536(3) Å for Ln = Ce vs 4.6157(4) Å for Ln = Nd.

**Figure 1:**
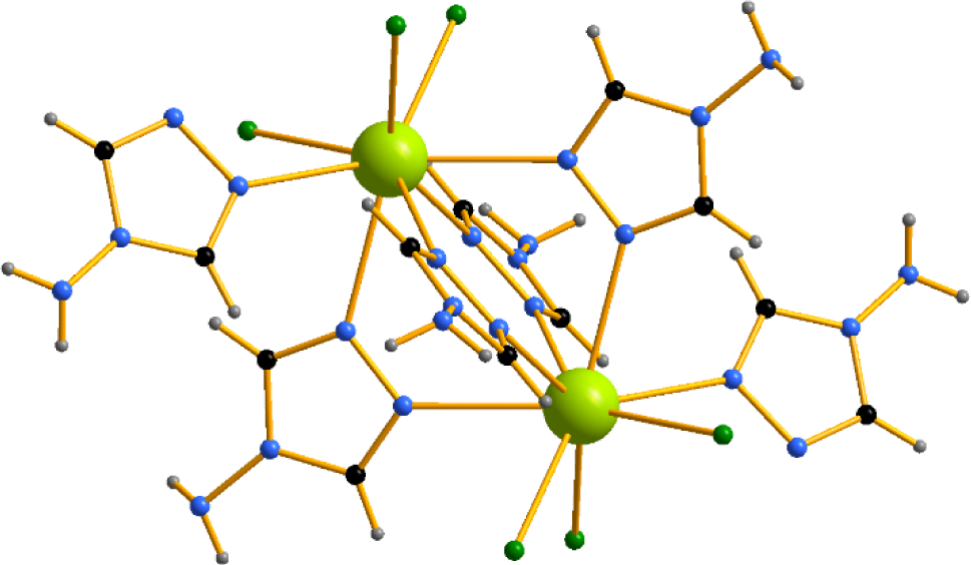
Dinuclear Nd complex in the crystal structure of [Nd_2_Cl_6_(μ_2_-4-NH_2_-1,2,4-Triaz)_4_(4-NH_2_-1,2,4-Triaz)_2_]. Nd atoms are light green, C – black, N – blue, Cl – dark green and H – gray.

The complexes noted above are discrete dimers, terminated by an “extra” azole ligand coordinating in a monodentate fashion. We have also been able to isolate a related anhydrous coordination polymer where bridging Cl^–^ is found without the terminating azole, [Ce_2_Cl_4_(μ_2_-Cl)_2_(μ_2_-4-NH_2_-1,2,4-Triaz)_4_]_n_ (*P*2_1_/*n*, *a* = 8.3938(3), *b* = 16.2647(6), *c* = 9.1975(3) Å, β = 106.646(4) °, *V* = 1203.05(8) Å^3^, Z = 2). Here again, two different products, this unhydrolyzed product and hydrolyzed [Ce_2_(μ_2_-Cl)_4_(μ_2_–OH)_2_(μ_2_-4-NH_2_-1,2,4-Triaz)_2_]_n_ were isolated at the same time from the same reaction mixture.

Similarly to [Ce_2_Cl_6_(μ_2_-4-NH_2_-1,2,4-Triaz)_4_(4-NH_2_-1,2,4-Triaz)_2_],^13^ [Ce_2_Cl_4_(μ_2_-Cl)_2_(μ_2_-4-NH_2_-1,2,4-Triaz)_4_]_n_ consists of dimeric units bound via four azole bridges. However, these dimeric units are not well separated and form additional bridges via two coordinated Cl^–^ ions and two CH···Cl^–^ hydrogen bonds stacking along the *a* axis (Figure 2). The Ce–Cl distances involved in the bridging are somewhat longer, 2.8639–2.9195(5) Å, compared to the terminal Ce–Cl, 2.7675–2.8053(6) Å. The CH···Cl^–^ hydrogen bonds between the aromatic rings and the terminal Cl^–^ ligands are 2.6131 (5) Å (dashed blue lines). The polymeric chains in turn stack in a hexagonal-like arrangement (Figure 3) bound via longer NH···Cl^–^ hydrogen bonds (*d*_Cl–H_ > 2.75 Å) and additionally NH···π connectivities between the azole aromatic rings and the neighboring NH_2_ azole groups (*d*_Cg‑H_ ≥ 3 Å).

**Figure 2:**
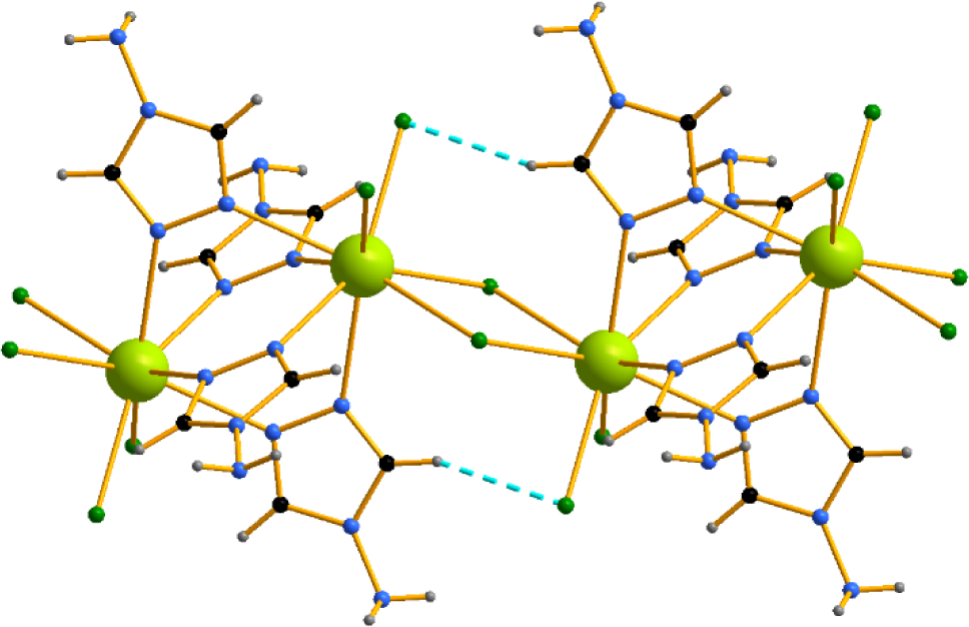
Dinuclear [Ce_2_Cl_4_(μ_2_-Cl)_2_(μ_2_-4-NH_2_-1,2,4-Triaz)_4_]_n_ complexes forming coordination polymers via Cl^–^ bridging and further stacking into a 1D coordination polymer.

**Figure 3:**
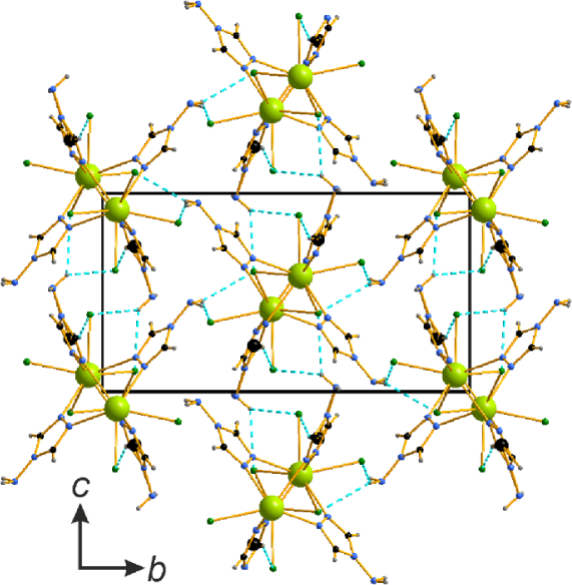
Hexagonal stacking of the dinuclear polymers [Ce_2_Cl_4_(μ_2_-Cl)_2_(μ_2_-4-NH_2_-1,2,4-Triaz)_4_]_n_ in the crystal packing.

It is clearly possible to isolate common coordination complexes using the 4-NH_2_-1,2,4-Triaz ligand, but it is perhaps the isolation of such a wide range of hydrolysis products that highlights the usefulness of this ligand. Utilizing essentially the same reaction conditions has allowed us to isolate what we consider to be the lowest degree of hydrolysis, [Ce_2_(μ_2_-Cl)_4_(μ_2_-OH)_2_(μ_2_-4-NH_2_-1,2,4-Triaz)_2_]_n_ (*Cmca*, *a* = 15.4096(5), *b* = 7.3602(3), *c* = 13.3266(5) Å, *V* = 1511.5(1) Å^3^, Z = 2). In this structure, two Ce atoms are bound via two μ_2_-4-NH_2_-1,2,4-Triaz ligands and two OH^–^ bridges (Figure 4). The Ce–O­(H) distances are 2.451–2.466(5) Å, suggesting that the Ce­(III) has not been oxidized and remains trivalent. Here, all terminal Cl^–^ ligands are shared between two such units serving as bridges (d_Ce–Cl_ = 2.895–2.899(1) Å) to form polymeric layers (Figure 5) in the bc plane. In each layer, each dimeric unit is bound via Cl^–^ bridges to four identical ones resulting in a slightly distorted hexagonal pattern of the cationic part. On the other hand, the layers stack along the *a* axis via pretty strong hydrogen bonding between the azole NH_2_ groups and OH^–^ bridges (d_NH···O(H)_ = 1.96 Å), and parallel π···π stacking of the aromatic azole rings (Figure S1, d_Cg···Cg_ = 3.8247(1) Å, d_Cg···N_ = 3.6814(2) Å).

**Figure 4:**
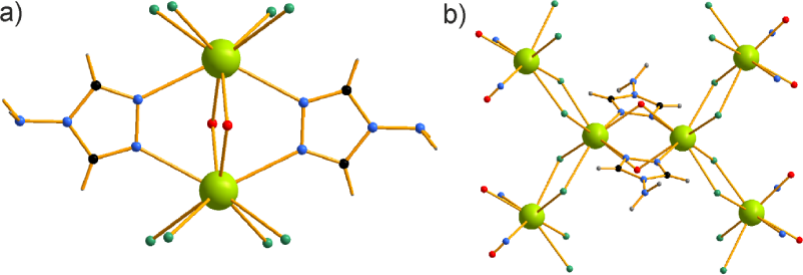
[Ce_2_(μ_2_-Cl)_4_(μ_2_–OH)_2_(μ_2_-4-NH_2_-1,2,4-Triaz)_2_]_n_ complex (a) and (b) connectivities to four neighboring complexes via shared Cl^–^ ligands.

**Figure 5:**
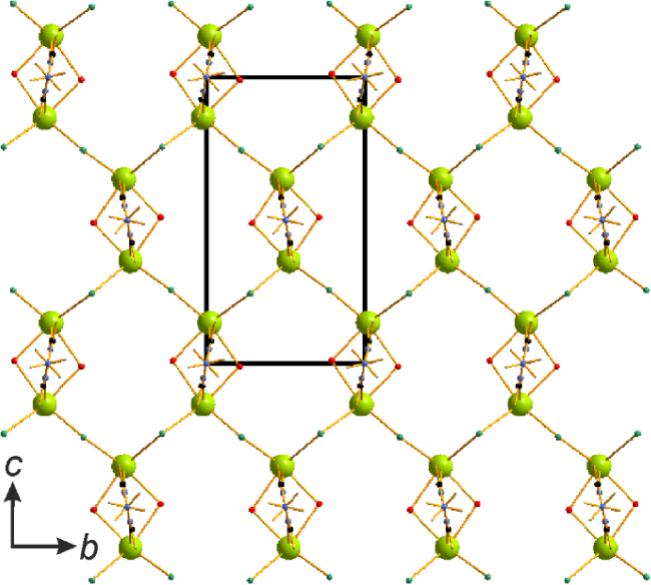
Packing in the crystal structure of [Ce_2_(μ_2_-Cl)_4_(μ_2_-OH)_2_(μ_2_-4-NH_2_-1,2,4-Triaz)_2_]_*n*_.

From the same reaction vessel which provided [Nd_2_Cl_6_(μ_2_-4-NH_2_-1,2,4-Triaz)_4_(4-NH_2_-1,2,4-Triaz)_2_], we were also able to isolate the hydrolyzed tetramer [Nd_4_Cl_4_(μ_2_-Cl)_4_(μ_3_-OH)_4_(μ_2_-4-NH_2_-1,2,4-Triaz)_4_]_*n*_·2*n*H_2_O with a similar degree of hydrolysis and structure building principles. The isostructural Ce­(III) compound was isolated using similar reaction conditions. [Ln_4_Cl_4_(μ_2_-Cl)_4_(μ_3_-OH)_4_(μ_2_-4-NH_2_-1,2,4-Triaz)_4_]_*n*_·2*n*H_2_O (Ln = Ce, Nd) crystallize in the *I*4_1_/*a* space group with a = 21.0186(4), c = 7.2994(2) Å, V = 3224.7(2) Å^3^, Z = 4 for Ce and a = 20.9319(4), c = 7.2438(2) Å, V = 3173.8(2) Å^3^, Z = 4 for Nd. Each three Ln­(III) atoms are pairwise bonded via four μ_3_-OH^–^ groups forming tetrahedral stars and additionally via four μ_2_-4-NH_2_-1,2,4-Triaz ligands (Figure 6). Two out of six Ln_4_ tetrahedron edges are not connected in this way, although this may rather be related to the peculiarities of the polymer formation rather than to the hydrolysis directly. The Ln–Ln edges not connected via 4-NH_2_-1,2,4-Triaz ligands are slightly shorter 3.9854(6) vs 3.9945(4) Å for Ce and 3.9305(6) vs 3.9467(4) Å for Nd. All Ln–O contacts are 2.483(1) (Ce) and 2.453(1) (Nd) Å. For Ln = Nd, these are on average slightly shorter compared to Ce–O contacts in other Ce compounds in this work following the lanthanide contraction. We would note that this is additional support for having maintained the Ce­(III) oxidation state.

**Figure 6:**
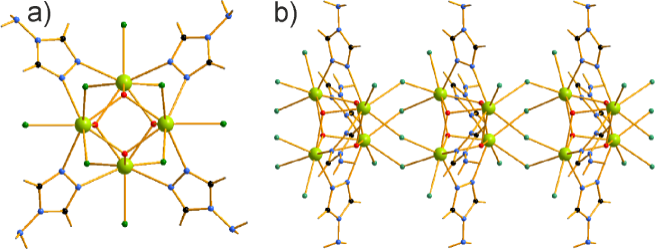
[Ln_4_Cl_4_(μ_2_-Cl)_4_(μ_3_-OH)_4_(μ_2_-4-NH_2_-1,2,4-Triaz)_4_]_*n*_·2*n*H_2_O (Ln = Ce, Nd—shown) (a) and (b) a portion of the polymeric chain extending along the *c* axis.

In contrast to [Ce_2_(μ_2_-Cl)_4_(μ_2_-OH)_2_(μ_2_-4-NH_2_-1,2,4-Triaz)_2_]_n_, the μ_2_-Cl^–^ bridges between the [Ln_4_Cl_4_(μ_2_-Cl)_4_(μ_3_-OH)_4_(μ_2_-4-NH_2_-1,2,4-Triaz)_4_]_*n*_·2*n*H_2_O (Ln = Ce, Nd) complexes expand solely along one direction, forming columns. The connectivity between the columns is established exclusively via π···π stacking with *d*_Cg‑C_ = 3.381(2) Å. Each complex is bound to four neighboring complexes, establishing square tunnels (Figure 7). The tunnels are occupied by occupationally disordered water molecules forming helices along the *c* direction. Interestingly, Cl···H hydrogen bonding is observed solely between the Cl^–^ ligands and disordered water molecules in these tunnels.

**Figure 7:**
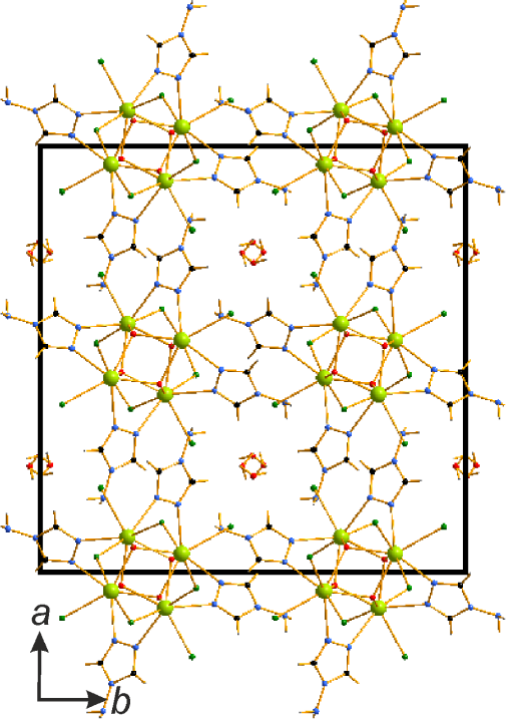
Packing in the crystal structure of [Nd_4_Cl_4_(μ_2_-Cl)_4_(μ_3_–OH)_4_(μ_2_-4-NH_2_-1,2,4-Triaz)_4_]_*n*_·2*n*H_2_O.

Our current study also isolated one, rather complex hexamer hydrolysis product, [Ce_6_Cl_6_(μ_6_-O_0.5_)­(μ_3_-Cl_0.5_)_4_(μ_3_-Cl_0.75_)_3_(μ_3_-OH)_0.75_(μ_2_-4-NH_2_-1,2,4-Triaz)_12_((OH_2_)_0.25_)_2_]_2_[CeCl_6_]­[Cl_9_]·*x*H_2_O (*P*3̅, a = 18.1657(8), c = 14.1649(8) Å, V = 4048.1(4) Å^3^, Z = 1). This complex, disordered compound adds to our growing list of such disordered species including [Ce_6_(μ_3_-O)_4_(μ_3_–OH)_2_(μ_3_-Cl)_2_Cl_6_(μ_2_-4-NH_2_-1,2,4-Triaz)_12_]·7H_2_O,^13^ [Nd_6_(μ_3_-OH)_8_Cl_6_(μ_2_-4-NH_2_-1,2,4-Triaz)_12_]­[Cl_4_]·2H_2_O, [Eu_6_(μ_6_-Cl)_0.23_(μ_3_-O_0.77_)_4_(μ_3_-O)_2.6_(μ_3_-Cl)_0.4_Cl_6_(μ_2_-4-NH_2_-1,2,4-Triaz)_12_], [Ho_6_(μ_6_-Cl)_0.21_(μ_3_-O_0.79_)_4_(μ_3_-OH)_2_Cl_6_(μ_2_-4-NH_2_-1,2,4-Triaz)_12_]­[Cl]_3.4_, and [Ce_6_(μ_3_–OH)_8_(BrPbBr_5_)­(μ_2_-4-NH_2_-1,2,4-Triaz)_11.5_(OH_2_)_6_]­[Pb_0.84_Br_4.2_]­[Br]_3.8_·2­(4-NH_2_-1,2,4-Triaz)·3.6H_2_O.^14^

[Ce_6_Cl_6_(μ_6_-O_0.5_)­(μ_3_-Cl_0.5_)_4_(μ_3_-Cl_0.75_)_3_(μ_3_–OH)_0.75_(μ_2_-4-NH_2_-1,2,4-Triaz)_12_((OH_2_)_0.25_)_2_]_2_[CeCl_6_]­[Cl_9_]·*x*H_2_O is based on a mixture of Ce_6_X_8_ and a rare Ce_6_X_9_ hexanuclear core,^19^ though with unique occupational and positional disorder. In our recent review of 4f and 5f M_6_O_8_ and M_6_O_9_ hydrolysis products, of the 184 unique such compounds found in the Cambridge Crystallographic Database (CSD)^20^ or Inorganic Crystal Structure Database (ICSD, FIZ Karlsruhe), only 76 contained 4f elements with 31 of those containing Ce­(IV) studied for its catalytic behavior. There was only one Ce­(III) example and that was our recently reported compound noted earlier. Of the 76 4f compounds, only 24 included a μ_6_-O^2–^ anion (i.e., M_6_O_9_) and these were exclusively Ln­(III) compounds with no examples of Ce­(III/IV). In the synthesis we report here, we have captured a mixture of Ce­(III) in both the Ce_6_X_8_ (second example isolated) and Ce_6_X_9_ (first example isolated) forms. This compound also represents only the third example of a non-μ_3_-O^2–^ or μ_3_–OH^–^ occupying the inner core of any of the 184 M_6_O_8_ or M_6_O_9_ compounds, the other examples being our Ce­(III) [Ce_6_(μ_3_-O)_4_(μ_3_–OH)_2_(μ_3_-Cl)_2_Cl_6_(μ_2_-4-NH_2_-1,2,4-Triaz)_12_]·7H_2_O and the previously reported Ce­(IV) [Ce_6_(μ_3_-O)_4_(μ_3_-OH)_3_(μ_3_-F)­(μ_2_-benzoate)_12_(NC_5_H_5_)_2_].^21^

[Ce_6_Cl_6_(μ_6_-O_0.5_)­(μ_3_-Cl_0.5_)_4_(μ_3_-Cl_0.75_)_3_(μ_3_–OH)_0.75_(μ_2_-4-NH_2_-1,2,4-Triaz)_12_((OH_2_)_0.25_)_2_]_2_[CeCl_6_]­[Cl_9_]·*x*H_2_O, however, is even more complex than its disordered inner core composition, consisting of two principally different metal components—hexanuclear and mononuclear (Figure 8). The metal centers of the hexanuclear core are maximally 9-coordinated and display a distorted capped square antiprismatic geometry. The coordination number is, however, affected by the occupation of the μ_6_- and μ_3_-positions. All metal centers are coordinated to four bridging μ_2_-4-NH_2_-1,2,4-Triaz ligands and a terminal Cl^–^ position. Within the oxide core, they coordinate to either four μ_3_-Cl^–^/O^2–^ or two μ_3_-Cl^–^/O^2–^ and one μ_6_-O^2–^_0.5_. It is worth noting that Ce–O distances (all >2.6 Å) are clearly indicative of the Ce­(III) valence state.^19^ The Ce_6_ octahedron shows minor disorder with the Ce–Ce distances being in a narrow range of 4.1328–4.1563(4) Å and the Ce–Ce–Ce angles within the triangular faces do not exceed 60.0(3)°. The Ce–Ce distances also vary slightly—2.826(2) and 2.844(2) Å. The mononuclear component is represented by a fully ordered octahedral [CeCl_6_]^3–^ with all Ce–Cl distances = 2.756(3) Å, while the Cl–Cl–Cl angles show a little higher deviation—60(1)°.

**Figure 8:**
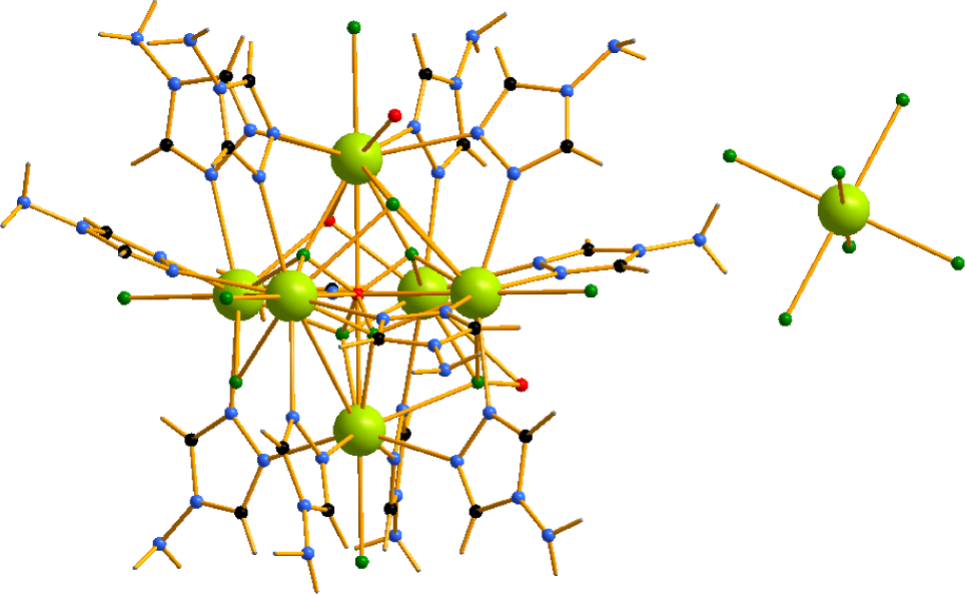
[Ce_6_Cl_6_(μ_6_-O_0.5_)­(μ_3_-Cl_0.5_)_4_(μ_3_-Cl_0.75_)_3_(μ_3_-OH)_0.75_(μ_2_-4-NH_2_-1,2,4-Triaz)_12_((OH_2_)_0.25_)_2_]_2_[CeCl_6_]­[Cl_9_]·*x*H_2_O.

The crystal packing is characterized by the hexanuclear complexes forming zigzag chains along the *c* axis segregating the mononuclear complexes (Figure 9). All connectivities between the complexes are established via an NH_2_···Cl^–^ hydrogen bonding network. Each hexanuclear complex is surrounded by six identical ones in a trigonal prismatic arrangement and three mononuclear centering prism faces. Cl···H contacts start from 2.38 Å and two such contacts are formed between each two hexanuclear complexes, while just one bond is observed between the hexa- and mononuclear complexes. The intercluster space contains additional disordered chloride anions for charge balance and, perhaps, disordered water. Some of the chloride anions center the cups formed by three triazole ligands, an observation that is typical for similar complexes perhaps due to lone-pair-π connectivities.

**Figure 9:**
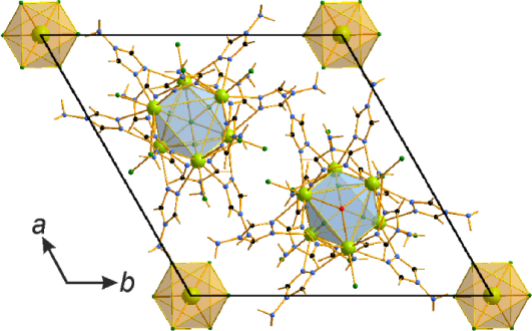
Packing in the crystal structure of [Ce_6_Cl_6_(μ_6_-O_0.5_)­(μ_3_-Cl_0.5_)_4_(μ_3_-Cl_0.75_)_3_(μ_3_-OH)_0.75_(μ_2_-4-NH_2_-1,2,4-Triaz)_12_((OH_2_)_0.25_)_2_]_2_[CeCl_6_]­[Cl_9_]·*x*H_2_O. Hexanuclear complexes are highlighted blue and mononuclear in orange.

## Conclusions

The current work offers important insights into capturing structural snapshots of f-element hydrolysis products within a chemically coherent and controllable melt environment. Unlike aqueous systems, which often lead to rapid precipitation or poorly defined colloids, the azole melt used here functions as both ligand source and solvent, enabling the stepwise and reproducible isolation of well-defined complexes across a hydrolysis spectrum from anhydrous monomers and dimers to fully hydrolyzed hexanuclear clusters. The 4-amino-1,2,4-triazole ligand used in this work exhibits a unique ability to stabilize diverse nuclearities and guide the formation of structurally rich assemblies, a feature rarely observed in similar ligand systems. In addition, the simultaneous isolation of hydrolyzed and nonhydrolyzed products from the same reaction conditions provides rare direct evidence of intermediates along the hydrolytic pathway, giving this work mechanistic depth that goes beyond typical coordination studies.

In the course of our study, we were able to “capture snapshots” of different stages toward the formation of the octahedral hexanuclear N–N bound lanthanide complexes, uncovering a broad diversity of intermediate stages. At the earlier stages, azole molecules bind two Ln centers via partial substitution of the chloride ligands, expelling water molecules, resulting in an isolated dinuclear complex [Nd_2_Cl_6_(μ_2_-4-NH_2_-1,2,4-Triaz)_4_(4-NH_2_-1,2,4-Triaz)_2_] or polymerization of the dimer via common Cl^–^ ligands as in [Ce_2_Cl_4_(μ_2_-Cl)_2_(μ_2_-4-NH_2_-1,2,4-Triaz)_4_]_n_ and partial hydrolysis combined with polymerization, such as observed in the formation of [Ce_2_(μ_2_-Cl)_4_(μ_2_-OH)_2_(μ_2_-4-NH_2_-1,2,4-Triaz)_2_]_n_ based on dimeric units and [Ln_4_Cl_4_(μ_2_-Cl)_4_(μ_3_-OH)_4_(μ_2_-4-NH_2_-1,2,4-Triaz)_4_]_*n*_·2*n*H_2_O based on tetrameric units. In the hydrolysis products, hydroxide ions are involved in the complex core formation, while additional water molecules may be present in the voids. Finally, we could even observe formation of the more heavily hydrolyzed hexanuclear core together with untouched [CeCl_6_][Bibr ref3]– in [Ce_6_Cl_6_(μ_6_-O_0.5_)­(μ_3_-Cl_0.5_)_4_(μ_3_-Cl_0.75_)_3_(μ_3_–OH)_0.75_(μ_2_-4-NH_2_-1,2,4-riaz)_12_((OH_2_)_0.25_)_2_]_2_[CeCl_6_]­[Cl_9_]·*x*H_2_O. As we often find in the hexameric hydrolysis products, the resulting Ce_6_X_8_/Ce_6_X_9_ core is highly disordered representing several forms which cocrystallize giving the average formulation which was crystallographically determined.

While our experiments employed commercially available hydrated lanthanide salts and were conducted under ambient atmospheric conditions, we successfully isolated and structurally confirmed anhydrous products. This suggests that the 4-amino-1,2,4-triazole ligand and azole melt medium are highly effective at displacing water from the metal coordination sphere. Nonetheless, we acknowledge that conducting the synthesis under rigorously anhydrous and anaerobic conditions with predried reagents may further favor the formation of exclusively nonhydrolyzed species. Although this was not the goal of our present work (i.e., the study of hydrolysis intermediates), future studies will explore this synthetic route to better control product selectivity and assess the impact of reaction conditions on hydrolysis suppression. Once these conditions are established, it would be beneficial to determine if the hydrolyzed products are formed in a controlled manner when stoichiometric amounts of water are added.

The 4-amino-1,2,4-triazole (4-NH_2_-1,2,4-Triaz) ligand plays an essential role in directing the hydrolysis behavior of lanthanide­(III) ions under azole melt conditions. Its ability to coordinate via multiple nitrogen atoms enables the early stabilization of metal ions, forming discrete or polymeric nonhydrolyzed species that delay uncontrolled precipitation. In this prehydrolytic stage, 4-NH_2_-1,2,4-Triaz acts as a primary coordinating agent, displacing water molecules from the metal coordination sphere, effectively buffering the onset of hydrolysis. As the reaction progresses, hydrolysis begins to occur in a more controlled and stepwise fashion, with the ligand maintaining structural integrity by bridging adjacent metal centers. This results in the ordered formation of higher-nuclearity species such as dimers, tetramers, and hexamers, where hydroxide or oxide ligands are incorporated into stable, ligand-supported cores. Furthermore, the templating nature of the 4-NH_2_-1,2,4-Triaz ligand guides the overall topology of the resulting clusters and facilitates crystallization of intermediate hydrolyzed fragments that would otherwise remain elusive or amorphous. Thus, the ligand not only mitigates uncontrolled hydrolysis but also enables the structural capture of dynamic species along the hydrolytic pathway.

Although symmetric polynuclear Ln_6_X_8_ and Ln_6_X_9_ units are common,^19^ the majority of the products we observe with 4-NH_2_-1,2,4-Triaz show an incomplete octahedral core with either the μ_6_- or some μ_3_-positions being partially occupied or completely missing. For instance, while the complex with Nd, [Nd_6_(μ_3_-OH)_8_Cl_6_(μ_2_-4-NH_2_-1,2,4-Triaz)_12_]­[Cl_4_]·2H_2_O, is highly regular, those with Eu ([Eu_6_(μ_6_-Cl)_0.23_(μ_3_-O_0.77_)_4_(μ_3_-O)_2.6_(μ_3_-Cl)_0.4_Cl_6_(μ_2_-4-NH_2_-1,2,4-Triaz)_12_]) or Ho ([Ho_6_(μ_6_-Cl)_0.21_(μ_3_-O_0.79_)_4_(μ_3_-OH)_2_Cl_6_(μ_2_-4-NH_2_-1,2,4-Triaz)_12_]­[Cl]_3.4_) are significantly distorted, with both μ_6_- and μ_3_-shells being significantly underoccupied.^14^ It is also worth noting that a very limited number of compounds with the M_6_O_9_ core has been reported and those are solely with trivalent lanthanides, even though there are many more crystal structures containing Ce­(IV) or actinides (all of which are the M_6_O_8_ form). Also, in contrast to previous findings with a potentially ambiguous Ce oxidation state, all compounds in this work contain solely trivalent Ce, as can be judged from the corresponding Ce–O and Ce–Cl distances.

As for lower-dimensional oligomers, e.g., dimers, trimers, or tetramers, in the literature beyond our previous work,^13^ their representation with N–N connectivity is limited to a few examples in the forms of Ln_2_ dimers,^22^ Ln_3_O triangles,^23^ double dimers bound via OH^–^ bridges,^24^ Ln_4_O tetrahedra,^25,26^ or Ln_4_O_4_ tetrahedra,^27^ although not necessarily containing azoles and showing little relationship to our current area. Although azoles can coordinate to the lanthanide ions in different coordination modes,^28,29^ particularly forming dimers,^30^ they are much harder to observe in larger oligomeric units, perhaps due to their bulky nature. On the other hand, these ligands are perfect linkers for the formation of MOFs offering the possibility to bind two, three, or even four metal centers and consequently leading to their large structural variety.^31^

In our review of crystal structures of lanthanide and actinide compounds in the M_6_X_8_ and M_6_X_9_ forms and in our own work in this area, we have found that the published crystallographic results are typically disordered in rather complex ways making it difficult to fully interpret the details of the bonding. In addition, the crystallographic disorder models that have been developed are subject to interpretation and methods to more fully resolve these species are needed. Although additional experimental techniques (e.g., spectroscopic tracking of solution dynamics or kinetic measurements) could further deepen the analysis, the ability to isolate and structurally characterize a wide variety of intermediates, including mono-, di-, tetra-, and hexanuclear species, already provides compelling evidence for the pathways proposed offering valuable insight into f-element coordination chemistry despite experimental challenges in obtaining single-phase products. Moreover, we believe that the combination of the synthetic approach together with X-ray crystallography constitutes a suitable methodology for the scope and aim of this study as individual snapshots we have obtained from intermediate phases seem quite clear and easy to model. We will continue to explore the last steps of the hydrolysis reactions to help tease out how these form in solution.

Looking forward, the insights gained from this study can directly inform the design of selective extractants and separation protocols for f-elements, particularly in the context of nuclear waste remediation and actinide-lanthanide separations. Moreover, the demonstrated ability to template high-nuclearity clusters with tailored ligand environments opens pathways toward new materials for catalysis, magnetism, and molecular electronics, especially where control over f-element nuclearity and connectivity is desirable. This work also establishes a platform for extending the approach to transuranic elements under similarly mild and ligand-directed conditions, offering potential value in actinide chemistry and materials science.

## Supplementary Material



## Notes

**Figure S1.** Layers stacking along the *a* axis in the crystal structure of [Ce_2_(μ_2_-Cl)_4_(μ_2_-OH)_2_(μ_2_-4-NH_2_-1,2,4-Triaz)_2_]_*n*_. **Tables S1.** Details of the crystal structure investigation and refinement. **Tables S2–S7.** Interatomic distances and angles in the coordination sphere of Ln.(PDF)

## #

Department of Chemistry and Nuclear Science and Engineering Center, Colorado School of Mines, 1500 Illinois St. Golden, CO, 80401, United States
