# Supplementary material for: Crystallographic Snapshots of Pre- and Post-Lanthanide Halide Hydrolysis—Reaction Products Captured by the 4‑Amino-1,2,4-triazole Ligand
Source: Cryst Growth Des. 2025 Jul 19;25(15):6421–8. doi: 10.1021/acs.cgd.5c00835 (PMC12368990; doi:10.1021/acs.cgd.5c00835)
Supplement: Supplementary file 1 [file cg5c00835_si_001.pdf]

Supplementary Information

Crystallographic Snapshots of pre- and post-Lanthanide Halide Hydrolysis - Reaction Products Captured by the 4-Amino-1,2,4-triazole Ligand

Volodymyr Smetana,[3] Geetha Bolla,[1,2] Ethan A. Hiti,[1] Hannah Wineinger,[2,†] Anja-Verena Mudring,[3,4*] and Robin D. Rogers[1,2*]

[1]*Department of Chemistry, University of Wyoming, 1000 E, University Ave, Laramie, WY 82071, United States. Email: robin.rogers@uwyo.edu.*

[2]*Department of Chemistry & Biochemistry, The University of Alabama, Tuscaloosa, AL, 35401 USA Email: rdrogers@ua.edu*

[3]*Department of Biological and Chemical Engineering and iNANO, Aarhus University, 8000 Aarhus C, Denmark. Email: anja-verena.mudring@bce.au.dk*

[4]*Department of Physics, Umeå University, 90187 Umeå, Denmark. Email: anja-verena.mudring@umu.se*

Content:

Figure S1. Layers stacking along the *a* axis in the crystal structure of $[Ce_2(\mu_2\text{-}Cl)_4(\mu_2\text{-}OH)_2(\mu_2\text{-}4\text{-}NH_2\text{-}1,2,4\text{-}Triaz)_2]_n$.
Table S1. Details of the crystal structure investigation and refinement.
Table S2. Interatomic distances and angles in the coordination sphere of Ce in $[Ce_2(\mu_2\text{-}Cl)_4(\mu_2\text{-}OH)_2(\mu_2\text{-}4\text{-}NH_2\text{-}1,2,4\text{-}Triaz)_2]_n$.
Table S3. Interatomic distances and angles in the coordination sphere of Ce in $[Ce_2Cl_4(\mu_2\text{-}Cl)_2(\mu_2\text{-}4\text{-}NH_2\text{-}1,2,4\text{-}Triaz)_4]_n$.
Table S4. Interatomic distances and angles in the coordination sphere of Ce in $[Ce_4Cl_4(\mu_2\text{-}Cl)_4(\mu_3\text{-}OH)_4(\mu_2\text{-}4\text{-}NH_2\text{-}1,2,4\text{-}Triaz)_4]_n\cdot 2nH_2O$.
Table S5. Interatomic distances and angles in the coordination sphere of Nd in $[Nd_4Cl_4(\mu_2\text{-}Cl)_4(\mu_3\text{-}OH)_4(\mu_2\text{-}4\text{-}NH_2\text{-}1,2,4\text{-}Triaz)_4]_n\cdot 2nH_2O$.
Table S6. Interatomic distances in the coordination sphere of Nd in $[Nd_2Cl_6(\mu_2\text{-}4\text{-}NH_2\text{-}1,2,4\text{-}Triaz)_4(4\text{-}NH_2\text{-}1,2,4\text{-}Triaz)_2]$.
Table S7. Interatomic distances and angles in the coordination sphere of Ce in $[Ce_6Cl_6(\mu_6\text{-}O_{0.5})(\mu_3\text{-}Cl_{0.5})_4(\mu_3\text{-}Cl_{0.75})_3(\mu_3\text{-}OH)_{0.75}(\mu_2\text{-}4\text{-}NH_2\text{-}1,2,4\text{-}Triaz)_{12}((OH_2)_{0.25})_2]_2[CeCl_6][Cl_9]\cdot xH_2O$.

† Present Address: Department of Chemistry and Nuclear Science and Engineering Center, Colorado School of Mines, 1500 Illinois St. Golden, CO, 80401

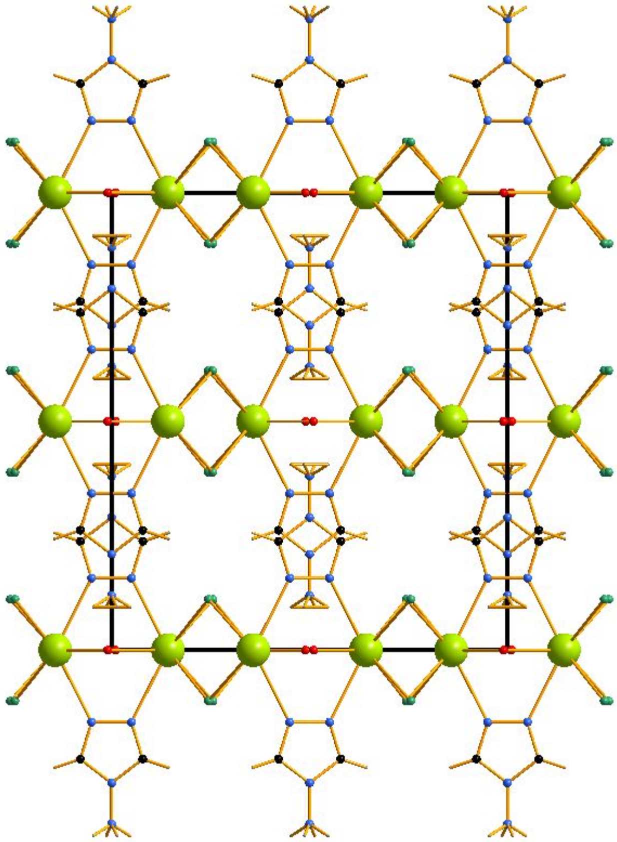

Figure S1. Layers stacking along the *a* axis in the crystal structure of $[Ce_2(\mu_2\text{-}Cl)_4(\mu_2\text{-}OH)_2(\mu_2\text{-}4\text{-}NH_2\text{-}1,2,4\text{-}Triaz)_2]_n$.

Table S1. Details of the crystal structure investigation and refinement.

	$[Ce_2(\mu_2\text{-}Cl)_4(\mu_2\text{-}OH)_2(\mu_2\text{-}4\text{-}NH_2\text{-}1,2,4\text{-}Triaz)_2]_n$	$[Ce_2Cl_4(\mu_2\text{-}Cl)_2(\mu_2\text{-}4\text{-}NH_2\text{-}1,2,4\text{-}Triaz)_4]_n$	$[Ce_4Cl_4(\mu_2\text{-}Cl)_4(\mu_3\text{-}OH)_4(\mu_2\text{-}4\text{-}NH_2\text{-}1,2,4\text{-}Triaz)_4]_n\bullet 2nH_2O$	$[Nd_4Cl_4(\mu_2\text{-}Cl)_4(\mu_3\text{-}OH)_4(\mu_2\text{-}4\text{-}NH_2\text{-}1,2,4\text{-}Triaz)_4]_n\bullet 2nH_2O$	$[Nd_2Cl_6(\mu_2\text{-}4\text{-}NH_2\text{-}1,2,4\text{-}Triaz)_4(4\text{-}NH_2\text{-}1,2,4\text{-}Triaz)_2]$	$[Ce_6Cl_6(\mu_6\text{-}O_{0.5})(\mu_3\text{-}Cl_{0.5})_4(\mu_3\text{-}Cl_{0.75})_3(\mu_3\text{-}OH)_{0.75}(\mu_2\text{-}4\text{-}NH_2\text{-}1,2,4\text{-}Triaz)_{12}((OH_2)_{0.25})_2]_2[CeCl_6][Cl_9]\bullet xH_2O$
CSD number	2454543	2454542	2454544	2454545	2454541	2454546
Empirical formula	$Ce_4C_8H_{16}N_{16}Cl_8O_4$	$Ce_2C_8H_{16}N_{16}Cl_6$	$Ce_4C_8H_{20}N_{16}Cl_8O_6$	$Nd_4C_8H_{20}N_{16}Cl_8O_6$	$Nd_2C1_2H_{24}N_{24}Cl_6$	$Ce_{13}C_{48}H_{96}N_{96}Cl_{32}O_4$
Formula weight	1244.45	829.31	1280.48	1296.96	1005.73	5044.25
Refinement method	Full-matrix least-squares on F^2					
Temperature	301(2)	300(2)	300(2)	300(2)	301(2)	100(2)
Radiation type	MoK$_\alpha$	MoK$_\alpha$	MoK$_\alpha$	MoK$_\alpha$	MoK$_\alpha$	MoK$_\alpha$
Wavelength, Å	0.71073	0.71073	0.71073	0.71073	0.71073	0.71073
Crystal system	orthorhombic	monoclinic	tetragonal	tetragonal	triclinic	trigonal
Space group	*Cmca*	$P2_1/n$	$I4_1/a$	$I4_1/a$	$P\bar{1}$	$P\bar{3}$
a, Å	15.4096(5)	8.3938(3)	21.0186(4)	20.9319(4)	9.1751(3)	18.1657(8)
b, Å	7.3602(3)	16.2647(6)	21.0186(4)	20.9319(4)	10.1310(4)	18.1657(8)
c, Å	13.3266(5)	9.1975(3)	7.2994(2)	7.2438(2)	17.4113(3)	14.1649(8)
		β = 106.646(4)°			α = 97.008(2)°<br>β = 92.681(2)°<br>γ= 90.710(3)°	
Volume, Å^3	1511.5(1)	1203.05(8)	3224.7(2)	3173.8(2)	1604.31(9)	4048.1(4)
Z	2	2	4	4	2	1
Density (calculated), g/cm3	2.734	2.289	2.637	2.715	2.082	2.067
μ, mm-1	6.665	4.436	6.256	7.163	3.751	4.168
F(000)	1152	788	2384	2417	972	2387
θ range	3.057 to 30.629º	2.504º to 30.699º	2.741 to 30.845º	1.946 to 30.719º	2.026 to 30.979º	2.664 to 29.000º
Index ranges	-19 < h < 19<br>-10 < k < 9<br>-18 < l < 18	-11 < h < 10<br>-20 < k < 23<br>-11 < l < 13	-29 < h < 25<br>-21 < k < 29<br>-8 < l < 9	-28 < h < 28<br>-25 < k < 25<br>-9 < l < 9	-12 < h < 11<br>-13 < k < 14<br>-22 < l < 24	-24 < h < 24<br>-24 < k < 24<br>-19 < l < 19
Reflections collected	3347	7945	6337	7519	22854	108212
Independent reflections	991	3022	2004	2038	8047	7156
Observed reflections	979	2950	1972	2010	7596	5956
Data/restraints/parameters	991/0/50	3022/0/147	2004/0/105	2038/0/105	8047/1/404	7156/20/339
Goodness-of-fit on F2	1.100	1.117	1.105	1.132	1.041	1.067
Final R indices [I>2σ(I)]	R1 = 0.0256<br>wR2 = 0.0679	R1 = 0.0148<br>wR2 = 0.0386	R1 = 0.0115<br>wR2 = 0.0312	R1 = 0.0119<br>wR2 = 0.0313	R1 = 0.0192<br>wR2 = 0.0504	R1 = 0.0449<br>wR2 = 0.0920
R (all data)	R1 = 0.0258<br>wR2 = 0.0681	R1 = 0.0153<br>wR2 = 0.0388	R1 = 0.0119<br>wR2 = 0.0313	R1 = 0.0123<br>wR2 = 0.0314	R1 = 0.0208<br>wR2 = 0.0511	R1 = 0.0558<br>wR2 = 0.0962
R_{int}	0.0190	0.0098	0.0091	0.0121	0.0180	0.0746
Largest diff. peak and hole, e–/Å3	1.965 and -1.539	0.582 and -0.634	0.689 and -0.333	0.786 and -0.519	0.810 and -0.830	2.097 and -2.924

Table S2. Interatomic distances and angles in the coordination sphere of Ce in $[Ce_2(\mu_2\text{-}Cl)_4(\mu_2\text{-}OH)_2(\mu_2\text{-}4\text{-}NH_2\text{-}1,2,4\text{-}Triaz)_2]_n$.

	distance (Å)		angle (°)
Ce-Cl	2.895(1)	Ce-O-Ce	101.3(3)
	2.899(1)	Ce-Cl-Ce	107.37(3)
Ce-N	2.717(4)	Ce-Ce-Ce	132.57(1)
Ce-O	2.451(6)	Ce-Ce-Ce	123.37(1)
	2.466(5)	Ce-Ce-Ce	104.05(1)
Ce-Ce	3.8031(4)		
	4.6684(4)		

Table S3. Interatomic distances and angles in the coordination sphere of Ce in $[Ce_2Cl_4(\mu_2\text{-}Cl)_2(\mu_2\text{-}4\text{-}NH_2\text{-}1,2,4\text{-}Triaz)_4]_n$.

	distance (Å)		angle (°)
Ce-Cl	2.7675(5)	Ce-Cl-Ce	108.06(2)
	2.8054(5)	Ce-Ce-Ce	130.00(1)
	2.8640(5)		
	2.9195(5)		
Ce-N	2.635(2)		
	2.655(2)		
	2.675(2)		
	2.670(2)		
Ce-Ce	4.5803(3)		
	4.6808(3)		

Table S4. Interatomic distances and angles in the coordination sphere of Ce in $[Ce_4Cl_4(\mu_2\text{-}Cl)_4(\mu_3\text{-}OH)_4(\mu_2\text{-}4\text{-}NH_2\text{-}1,2,4\text{-}Triaz)_4]_n\bullet 2nH_2O$.

	distance (Å)		angle (°)
Ce-Cl	2.8387(4)	Ce-Cl-Ce	131.52(2)
	2.8910(4)	Ce-O-Ce	108.07(4)
	2.9025(4)		107.67(4)
Ce-N	2.649(1)		107.14(4)
	2.652(1)	Ce-Ce-Ce	60.076(2)
Ce-O	2.465(1)		59.847(2)
	2.470(1)		
	2.483(1)		
Ce-Ce	3.9853(2)		
	3.9945(2)		
	3.9945(2)		

Table S5. Interatomic distances and angles in the coordination sphere of Nd in $[Nd_4Cl_4(\mu_2\text{-}Cl)_4(\mu_3\text{-}OH)_4(\mu_2\text{-}4\text{-}NH_2\text{-}1,2,4\text{-}Triaz)_4]_n \bullet 2nH_2O$.

	distance (Å)		angle (°)
Nd-Cl	2.8143(5)	Nd-Cl-Nd	131.64(2)
	2.8658(4)		107.93(5)
	2.8776(4)		106.87(5)
Nd-N	2.611(2)		107.50(4)
	2.612(2)	Nd-Nd-Nd	60.136(2)
Nd-O	2.440(1)		59.726(3)
	2.440(1)		
	2.453(1)		
Nd-Nd	3.9304(2)		
	3.9466(2)		
	3.9467(2)		

Table S6. Interatomic distances in the coordination sphere of Nd in $[Nd_2Cl_6(\mu_2\text{-}4\text{-}NH_2\text{-}1,2,4\text{-}Triaz)_4(4\text{-}NH_2\text{-}1,2,4\text{-}Triaz)_2]$.

	distance (Å)
Nd-Cl	2.7203(7)
	2.7221(6)
	2.8238(6)
	2.7120(6)
	2.7271(6)
	2.8097(5)
Nd-N	2.631(2)
	2.634(2)
	2.644(2)
	2.654(2)
	2.674(2)
	2.635(2)
	2.643(2)
	2.660(2)
	2.665(2)
	2.665(2)
Nd-Nd	4.6157(4)

Table S7. Interatomic distances and angles in the coordination sphere of Ce in $[Ce_6Cl_6(\mu_6\text{-}O_{0.5})(\mu_3\text{-}Cl_{0.5})_4(\mu_3\text{-}Cl_{0.75})_3(\mu_3\text{-}OH)_{0.75}(\mu_2\text{-}4\text{-}NH_2\text{-}1,2,4\text{-}Triaz)_{12}((OH_2)_{0.25})_2]_2[CeCl_6][Cl_9]\bullet xH_2O$.

	distance (Å)		angle (°)
Ce-Cl	2.404(3)	Ce-Cl-Ce	119.9(1)
	2.406(3)		118.7(1)
	2.826(1)		119.2(1)
	2.844(3)		119.29(4)
	2.844(1)	Ce-O-Ce	90.243(9)
	2.868(3)		89.596(9)
	2.958(3)		90.0(2)
	2.756(2)		90.0(2)
Ce-N	2.674(5)		179.52(13)
	2.676(5)	Ce-Ce-Ce	59.685(8)
	2.701(5)		60.000(9)
	2.712(5)		60.247(8)
	2.690(5)		89.839(9)
	2.705(5)		90.164(9)
	2.711(5)		
Ce-O	2.621(4)		
	2.90(1)		
	2.929(6)		
	2.935(6)		
Ce-Ce	4.1563(5)		
	4.1488(6)		
	4.1328(4)		
	4.1492(6)		
